# Staff and Facility Utilization in Direct Patient Transfer to the Comprehensive Stroke Center: Testing a Real-Time Location System for Automatic Patient Pathway Characterization

**DOI:** 10.3389/fneur.2021.741551

**Published:** 2021-11-24

**Authors:** Tiago Moreira, Alexander Furnica, Elke Daemen, Michael V. Mazya, Christina Sjöstrand, Magnus Kaijser, Evert van Loenen

**Affiliations:** ^1^Department of Neurology, Karolinska University Hospital, Stockholm, Sweden; ^2^Department of Clinical Neuroscience, Karolinska Institute, Stockholm, Sweden; ^3^Philips Research, Eindhoven, Netherlands; ^4^Department of Neurology, Danderyd Hospital, Stockholm, Sweden; ^5^Department of Neuroradiology, Karolinska University Hospital, Stockholm, Sweden

**Keywords:** triage, quality—hospital staff, logistics, nursing & care, telemedicine

## Abstract

**Introduction:** Starting reperfusion therapies as early as possible in acute ischemic strokes are of utmost importance to improve outcomes. The Comprehensive Stroke Centers (CSCs) can use surveys, shadowing personnel or perform journal analysis to improve logistics, which can be labor intensive, lack accuracy, and disturb the staff by requiring manual intervention. The aim of this study was to measure transport times, facility usage, and patient–staff colocalization with an automated real-time location system (RTLS).

**Patients and Methods:** We tested IR detection of patient wristbands and staff badges in parallel with a period when the triage of stroke patients was changed from admission to the emergency room (ER) to direct admission to neuroradiology.

**Results:** In total, 281 patients were enrolled. In 242/281 (86%) of cases, stroke patient logistics could be detected. Consistent patient–staff colocalizations were detected in 177/281 (63%) of cases. Bypassing the ER led to a significant decrease in median time neurologists spent with patients (from 15 to 9 min), but to an increase of the time nurses spent with patients (from 13 to 22 min; *p* = 0.036). Ischemic stroke patients used the most staff time (median 25 min) compared to hemorrhagic stroke patients (median 13 min) and stroke mimics (median 15 min).

**Discussion:** Time spent with patients increased for nurses, but decreased for neurologists after direct triage to the CSC. While lower in-hospital transport times were detected, time spent in neuroradiology (CT room and waiting) remained unchanged.

**Conclusion:** The RTLS could be used to measure the timestamps in stroke pathways and assist in staff allocation.

## Introduction

Stroke is a major source of morbidity and mortality and is now the second leading cause of death worldwide, accounting for 5.5 million deaths in 2016 (1). For every minute passing after a proximal middle cerebral artery occlusion without reperfusion therapy, 2 million brain neurons die (2). Therefore, when someone suffers from a stroke, it is critical to reach the hospital quickly and medical staff needs to act fast. For best functional outcome, an ischemic stroke victim with a large vessel occlusion needs to be treated in hospitals with access to endovascular thrombectomy (EVT) (3, 4).

The Karolinska University Hospital and the Stockholm Region developed and implemented the Stockholm Stroke Triage System, a three-step prehospital evaluation for primary stroke center (PSC) bypass for patients with suspected stroke and a moderate-to-severe hemiparesis. Bypass was initiated following teleconsultation between ambulance staff and a stroke physician at the Comprehensive Stroke Center (CSC) (5). Those approved for PSC bypass was directly transferred from the ambulance to the CSC department of neuroradiology, bypassing the emergency room (ER), if cardiorespiratory stable and not unconscious (5). The main goal of the Stockholm Stroke Triage System was to reduce the time from symptom onset to initiation of EVT without delaying intravenous thrombolysis (IVT), which is currently the central goal of acute stroke triage (6, 7). In parallel with the implementation of the new triage system, we conducted an observational study where the timestamps of the care pathway were automatically recorded by a real-time location system (RTLS), tracking patients and staff without direct observation by researchers and without interfering with the new pathway workflow. The principal aim of this study was to characterize the changes in logistics and staffing following PSC and ER bypass. We hypothesized that: (1) PSC and ER bypass of patients with severe symptoms to a CSC would result in an increase of the time spent by medical staff with more severely disabled patients (suspected to have large vessel occlusions) and (2) time patients spent at the neuroradiology department before a treatment decision for EVT was made would increase because initial medical assessment shifted from the ER to the neuroradiology unit. The impact of this study results could potentially be used for the changes in staffing and resource allocation.

## Patients and Methods

The study was conducted jointly by the Department of Neurology at the Karolinska University Hospital and by Philips Research between May 2017 and September 2018.

### Hardware Equipment

The RTLS system (CenTrak®, Newtown, Philadelphia, USA) based on IR and radiofrequency (RF) technology was installed at the Karolinska University Hospital in Solna (Supplementary Material, RTLS diagram). The choice of IR in detriment of other technologies such as low-energy based Wi-Fi, ultrasound, or Bluetooth was made to offer room-level accuracy, since signal leaking outside room range was more frequent in all the latter. The hardware system consists of tags, monitors, and virtual walls. Tags with IR sensors were applied to staff (badges) and patients (wrist bands), which interacted with monitors when they acquire line of sight. Monitors were placed on room ceilings and mapped to a location, so that when a tag reported to a specific monitor, the system could identify in which room it occurred. Finally, virtual walls acted similarly to monitors in that they were tied to a location; however, they were used to delimitate rooms.

### Patient Care Pathway Detection

To study and track the stroke patient pathway, IR monitors and virtual walls were installed in every location where a stroke patient would be transported. In total, 23 locations were equipped with monitors including ambulance bays, the ER, elevator halls, neuroradiology entry door, CT scan rooms, stroke unit entry door, and corridors where patients were transported. In addition, a wristband tag was placed on each patient and all the staff members were given a badge tag, which would report their location to the monitors. Wristbands were placed on patients at their arrival, if a stroke was suspected. The medical staff participating in this study consisted of neurologists, stroke nurses, neuroradiologists, and radiology nurses. Only staff instructed and delegated to participate in the study could include patients (about two-thirds of the stroke team workforce). The remaining workforce not participating in the study consisted of short term or shift workers who did not attend training or did not provide consent. Most often, a location would be enough in detecting a pathway step; however, there were some exceptions. For example, while the RTLS data indicate when a patient enters a CT room, it is unable to identify the specific procedures that occur inside the room, e.g., physical examination, administration of IVT. The RTLS had no connection to medical records of patients and, therefore, lacked context. To solve this challenge, some patient-level data were gathered in the form of a handwritten logbook filled by nurses along with manually retrieved electronic health record (EHR) data to double-check both the automatically generated RTLS data and the logbook. The main data points used for this purpose were the stroke type (ischemic, hemorrhagic) or if the patient had a stroke mimic and whether they were transferred from the ER or from another hospital (secondary transfer). The main pathways that the RTLS would cover were: (1) time elapsed between hospital arrival at ER or arrival at neuroradiology ambulance bay until exiting the CT room after imaging; (2) transport times between arrival point of entry and reaching the holding room at neuroradiology; (3) time spent waiting for a CT scan (on holding outside the CT room) and time elapsed in the CT room; (4) total time spent in the neuroradiology department (including time spent in CT scanning, clinical evaluation, IVT, and endovascular treatment at the neurointerventional suite); and (5) time measured when a nurse colocalized with the patient in the same room. The Department of Neuroradiology is equipped with two CT rooms and two conventional neuroendovascular rooms.

### Case Filtering Algorithm

The RTLS system continually reported every few seconds while a tag was moving and dropped down to every 5 min if the tag had been stationary for sometime. In addition, there was a period where the nurse was transporting the tag, which contained reports that were not relevant to the patient care pathway. To overcome this, we used a simple algorithm to filter out unnecessary data. First, we only kept tags that had a patient associated with them in the logbook and only the data for the day of patient arrival. Finally, we filtered out every report before one of the starting locations (either triage room or neuroradiology reception) was detected. Finally, we also checked the presence of the end location (elevators near neuroradiology reception) of the patient pathway. If all the conditions were met, the patient case was kept for further analysis.

### Analysis of Missing Data

Patient tags and staff badges always needed to be uncovered to be detected by IR sensors. Clothes, blankets, or other material sometimes obscured the tags and resulted in temporary loss of location. For instance, if CT room location was missing in one patient with a consistent stroke pathway, the patient was excluded from the “time in CT room” measurement.

### Phases of the Study

The trial was divided into two phases. Phase I ran from 1 May, 2017 until 10 October, 2017 and phase II started on 10 October, 2017, immediately after phase I, and ran until 31 September, 2018.

#### Baseline Pathway (Phase I)

**Figure 1:**
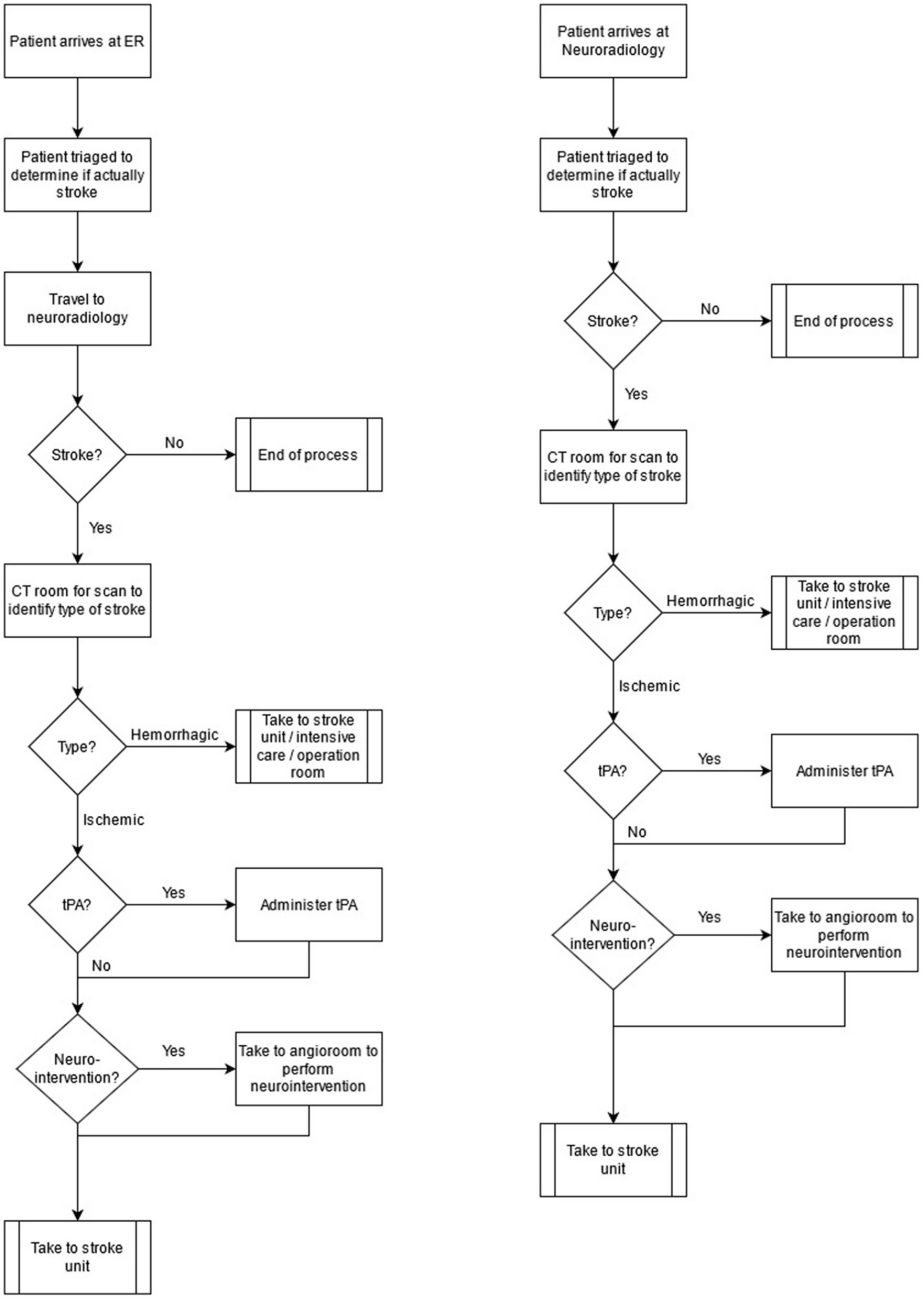
Stroke workflow, baseline (left) and new triage (right).

During baseline, the stroke care pathway was organized as follows (Figure 1, Left): the ambulance delivered all the suspected stroke patients to the ER after prehospital assessment using the Face, Arm, Speech, and Time (FAST) test without stroke physician prenotification. The neurologist on call (staffing the ER) immediately examined the patient to make an initial diagnosis at the ER including the National Institutes of Health Stroke Scale (NIHSS) assessment. Patient registration in the EHR, printing of ID tags, history taking, current medication check, vital signs, blood work (if indicated) and intravenous medication were also performed at the ER by a local nurse. An electrocardiogram (EKG) performed in the presence of angina pectoris was also performed by the local nurse. If the condition of the patient was assessed as a stroke, they were transported to the neuroradiology department passing two elevators and a corridor. Specific tasks for the stroke nurse at this stage were to collect personal belongings of the patient, assist with bed transport to neuroradiology, opening doors, move the patient to the CT board, communicate brief clinical information to the CT nurse, recheck the intravenous routes of the patient, change oxygen supply from portable to general, recheck vital signs at the CT room, calculate the dose for thrombolysis (alteplase) based on visual weight estimation, and prepare for and/or administer alteplase or intravenous medication when required. A CT scan was performed to determine the type of stroke. If the stroke was ischemic, then IVT was administered to eligible patients. CT angiography (CTA) and CT perfusion (CTP) were performed in all ischemic stroke patients to assess EVT indication and eligibility. During image postprocessing and multidisciplinary discussion between the neurologist, neuroradiologist, and interventional neuroradiologist, the patients and the stroke team moved to the neurointerventional suite on the same floor. Patients with hemorrhagic stroke were also evaluated with CTA unless contraindicated because of renal failure or in the presence of typical small vessel, small volume hemorrhage coincident with severe hypertension on arrival. Hemorrhagic stroke patients were either transferred directly to the stroke unit, to intensive care, or emergency neurosurgery, all located on the different floors. Patients assessed as having a stroke mimic in the ER would be referred to neuroradiology if their condition necessitated further radiological investigation (e.g., first seizure, suspected tumor) acutely or after admission to an in-patient ward.

#### New Triage Pathway (Phase II)

The stroke care pathway was reorganized as follows (Figure 1, Right): the ambulance nurses performed an initial assessment using the FAST test. If the patient was FAST test positive, an additional test was performed. If the patient scored ≥ 2 NIHSS points each in both the ipsilateral arm and leg (called the A2L2 test), the ambulance nurse teleconsulted the neurologist at the CSC. Conversely, in A2L2-negative cases, the ambulances prenotified the nearest PSC. During the CSC teleconsultation, the prehospital suspicion of stroke was confirmed, information on the premorbid level of function and comorbidities of patient was gathered, and a final triage decision was made, whether PSC bypass was indicated or not. Triage positive (A2L2 and teleconsult positive) cases were directly delivered to the neuroradiology department through a dedicated ambulance bay, eliminating ER assessment (otherwise conducted in a separate building). During ambulance transport, the stroke nurse at the stroke unit registered the patient in the EHR, printed ID tags, informed the ward team about the patient, prepared blood typing requests, checked the weight of the patient in the EHR and calculated the dose for thrombolysis, and loaded the pump for intravenous administration of thrombolysis. The neurologist checked current medication and previous medical history in the EHR. After patient arrival, vital signs, blood work, or EKG, when indicated, were then performed by the stroke nurse in the CT room or during holding time waiting for a CT scan. The neurologist examined the patient and performed the NIHSS assessment in the CT room. Specific tasks performed by the stroke nurse that followed in the CT room were then similar to the tasks in the baseline pathway. In both study phases, the neurologist and the stroke nurse were deployed to arrival of patient at the same time and the stroke nurse inserted a urinary catheter in all the patients selected for thrombectomy.

### Statistical Analysis

Descriptive statistics such as the one-way and two-way ANOVA with the Tukey's *post-hoc* tests were used to describe and detect the significant differences between the groups. Data are presented as median and interquartile ranges (IQRs). The RStudio software (Boston, MA, USA) was used for facility pathway analyses.

### Ethical Approval

This study was approved by the Stockholm Regional Ethical Council (nr 2017/511-31/4). Oral informed consent was obtained by a nurse or a physician immediately at arrival from patients without aphasia, able to understand the study information and to follow instructions. The wrist band was applied to the healthy arm. If able to use a pen, patients would sign the informed consent form as soon as possible and without delay to the acute stroke pathway. When written informed consent could not be obtained, the ethics approval still allowed the patients to be included in the study by the treating physician. Patients were contacted for providing their informed consent if they regained understanding of study consent during hospital stay. Staff members also provided informed consent for anonymized time and position tracking in the aforementioned locations.

### Patient and Public Involvement

Only patients were involved in this study, starting with May 7, 2017. In the informed consent form, patients were informed both orally and in writing, when able to read and sign, about the aims of the study. They also received information about the duration, individual risk/benefit of participation, handling of confidential personal data, insurance policy, and funding of the study. Thereafter, they also followed details on the technical aspects. The patients were also informed that they could stop participating in the study at any time. Neither patients nor the public were involved in the design and conduction of the study or the choice of outcome measures.

### Funding

The Philips Research Team provided hardware equipment, installation, and the RTLS data analysis.

## Results

The number of stroke code patients directly admitted from an ambulance to our stroke team was on average 350 patients per year during the study period. Over 17 months, a total of 281 patients were tagged with IR-sending wrist bands and in the EHR (55 patients in phase I and 226 patients in phase II). The total number of patients with acute ischemic stroke and intracerebral hemorrhage was 216 (77%) and 38 (13.4%), respectively. An additional 27 patients (9.6%) were stroke mimics (10 patients had epileptic seizures, four patients had migraine, two patients had tension headache, two patients had head trauma, two patients had confusion, and seven patients had other diagnoses). A total of 11 additional patients had been tagged with wrist bands, but not registered in the EHR and were, therefore, excluded.

### Occurrences Automatically Detected by the RTLS

The RTLS system could automatically detect 242 (86%) occurrences as possible stroke pathway patients. Of these, 65 (23%) had to be excluded related to the following: *system downtime* (5.7%)—the patient arrived during a period of the RTLS downtime when no movements were recorded, *likely a nurse* (5.3%)—the movement pattern in the data (e.g., multiple visits to the CT control room) suggested the tag was on a nurse rather than on the patient themselves, *missing data* (8.3%)—too much relevant data are missing from the pathway of patient. This could happen due to, for example, covering the tag with a blanket making the IR sensor unable to communicate with the monitors, and *data-log mismatch* (2.3%)—the RTLS data did not detect that a designated tag was attached to a patient or staff member, although being handwritten in the logbook. For example, on the supposed date of a patient having worn the tag, it was only detected in a single room. The total number of stroke pathway patients correctly detected by the RTLS was 177 (63% of all the tagged patients).

### Facility Pathway Analysis

#### Time Elapsed Between Hospital Arrival at ER or Neuroradiology Ambulance Bay Until Exiting the CT Room After Imaging (Arrival-To-CT Exit)

**Figure 2:**
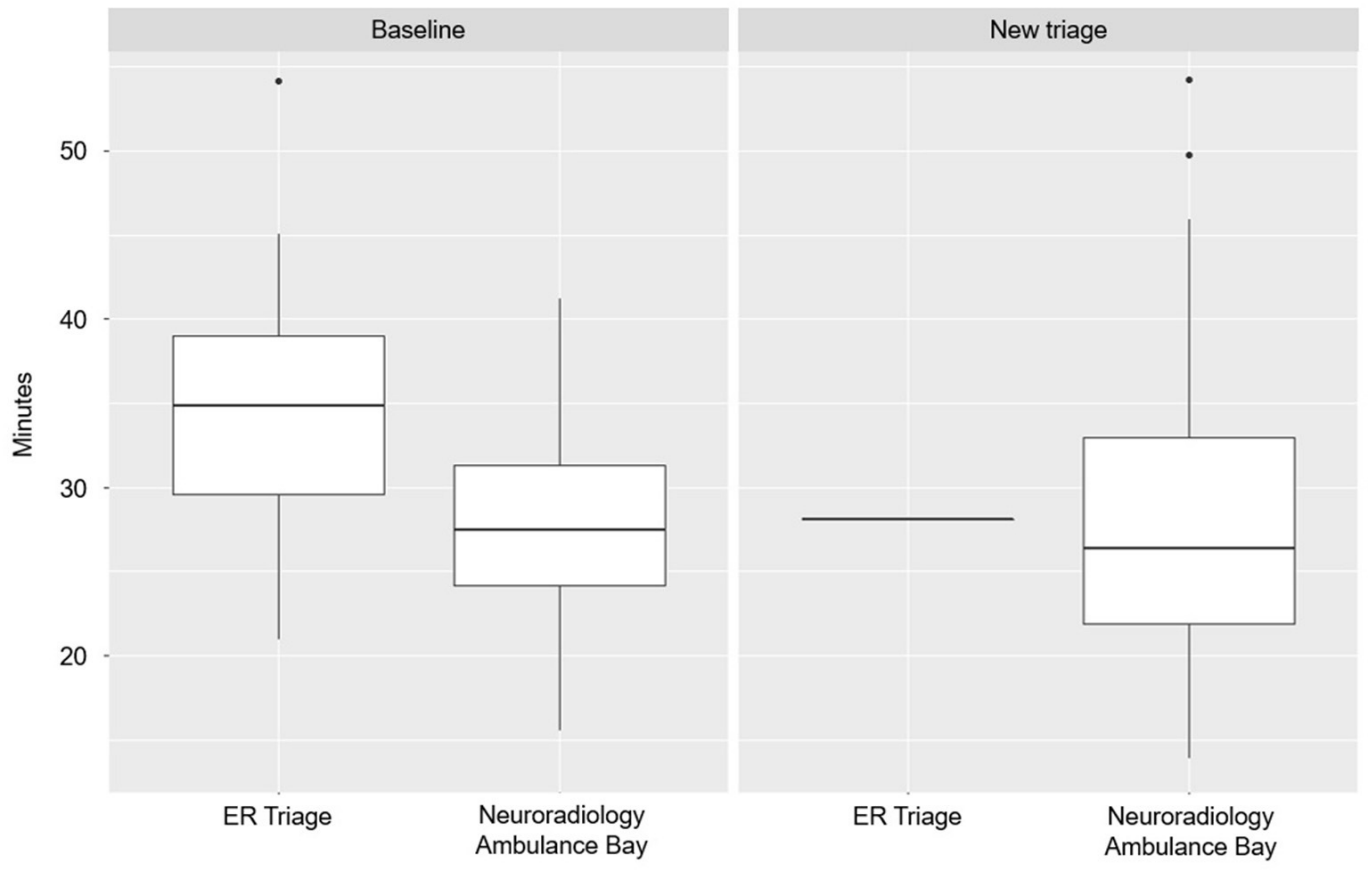
Time from hospital arrival to leaving the CT room comparing entry points (ER emergency room or neuroradiology ambulance bay).

At baseline, median time between hospital arrival to leaving the CT room was 35 min if patients were first admitted to the ER and 27.5 min if patients were admitted directly to neuroradiology (Figure 2). During the new triage system, all patients were admitted directly to neuroradiology with median time of hospital arrival-to-leaving the CT room of about 25 min.

#### Transport Times per Trial Phase

**Figure 3:**
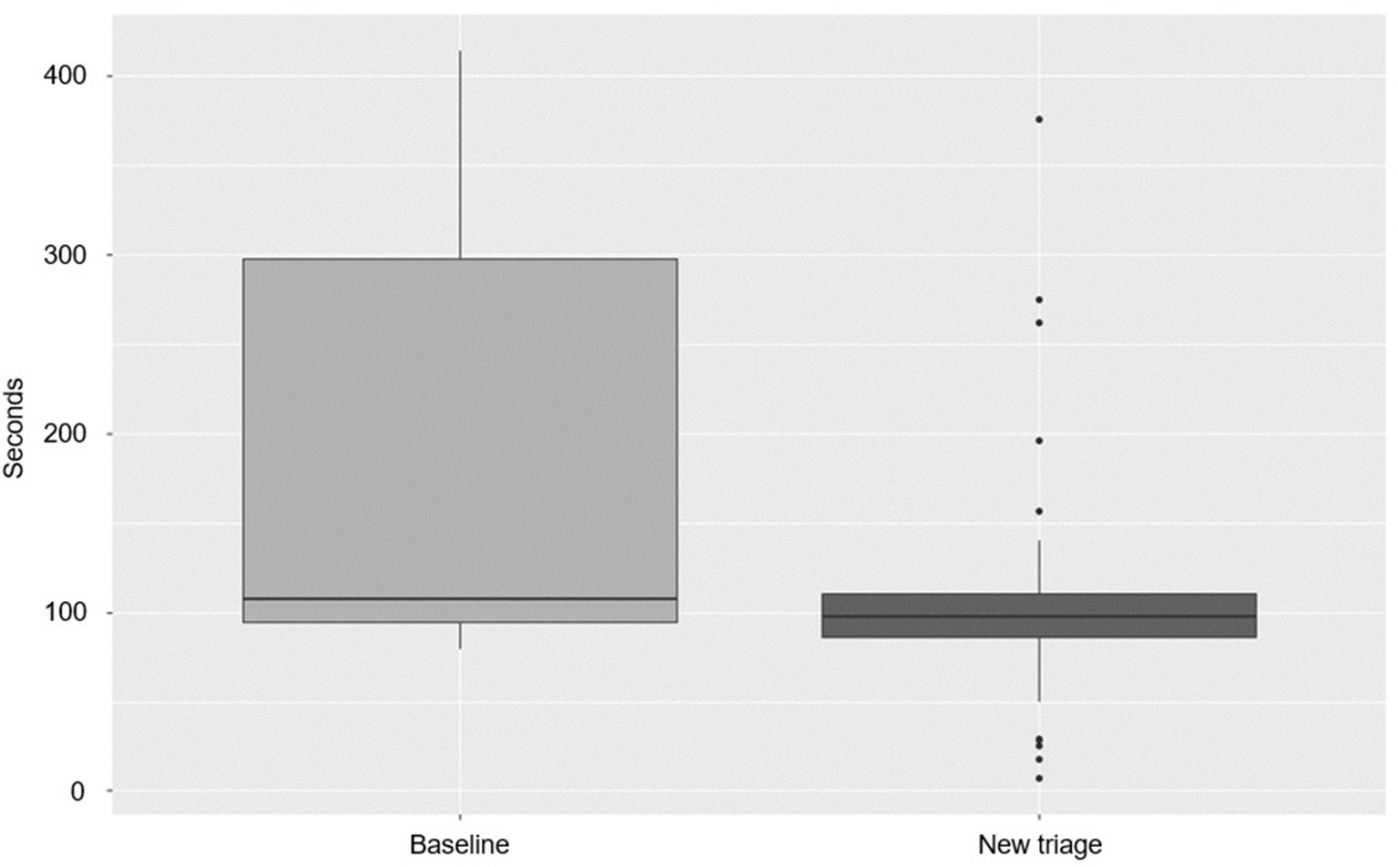
Transport time per study phase (*p* < 0.01).

The RTLS could measure the amount of time traveled through the hospital between leaving the arrival point of entry and reaching the holding room at neuroradiology (Figure 3).

There was a significant difference between the median baseline (107.5 s) and new triage (97.5 s) transport times (*p* < 0.01).

#### Holding and CT Room Times per Trial Phase and Stroke Type

In total, the RTLS detected time spent waiting before entering the CT room (time in holding), the time spent in the CT room, and the total time spent in neuroradiology (Table 1). There were no significant differences between time in holding and time in the CT room when baseline, new triage, and stroke types were compared. Patients presenting without stroke required the same amount of time as stroke patients.

**Table 1:** Time spent in holding in the CT room per study phase and stroke type, time spent in neuroradiology per stroke, and transfer types.

**Phase/Stroke type**	**Patients, *n***	**Median (IQR)**	***P* value**
*Time in holding (s)*			n/s
Baseline	29	13 (6)	0.813
New triage	71	13 (45)	0.813
Hemorrhagic stroke^*^	15	14 (3.5)	0.414
Ischemic stroke^*^	84	13 (6)	0.414
Stroke mimic^*^	13	13 (5)	0.414
*Time in CT room (min)*			n/s
Baseline	34	17.5 (8.3)	0.422
New triage	94	19.3 (9.3)	0.422
Hemorrhagic stroke	17	19.5 (7.9)	0.796
Ischemic stroke	113	17.4 (8.7)	0.796
Stroke mimic	13	16.9 (7.1)	0.796
*Time in neuroradiology (min)*			n/s
Hemorrhagic stroke	18	35.1 (17.3)	0.283
Ischemic stroke	108	50.6 (86.7)	0.283
Stroke mimic	13	38.7 (12.5)	0.283
Direct transfer	96	38.5 (35)	0.232
Secondary transfer	44	107.4 (103.6)	0.232

*IQR, interquartile range; n/s, non-significant.*

* *Time on holding per stroke type was compared after acquiring the postimaging diagnoses*.

#### Time in Neuroradiology per Stroke Type and in Direct vs. Secondary Transfer

Longer times spent in neuroradiology were observed for the ischemic strokes and patients arriving as secondary transfers from the primary stroke centers (Table 1), but not reaching statistical significance. Patients without stroke spent the same amount of time in neuroradiology as hemorrhagic stroke patients.

### Patient–Staff Pathway Analysis

**Figure 4:**
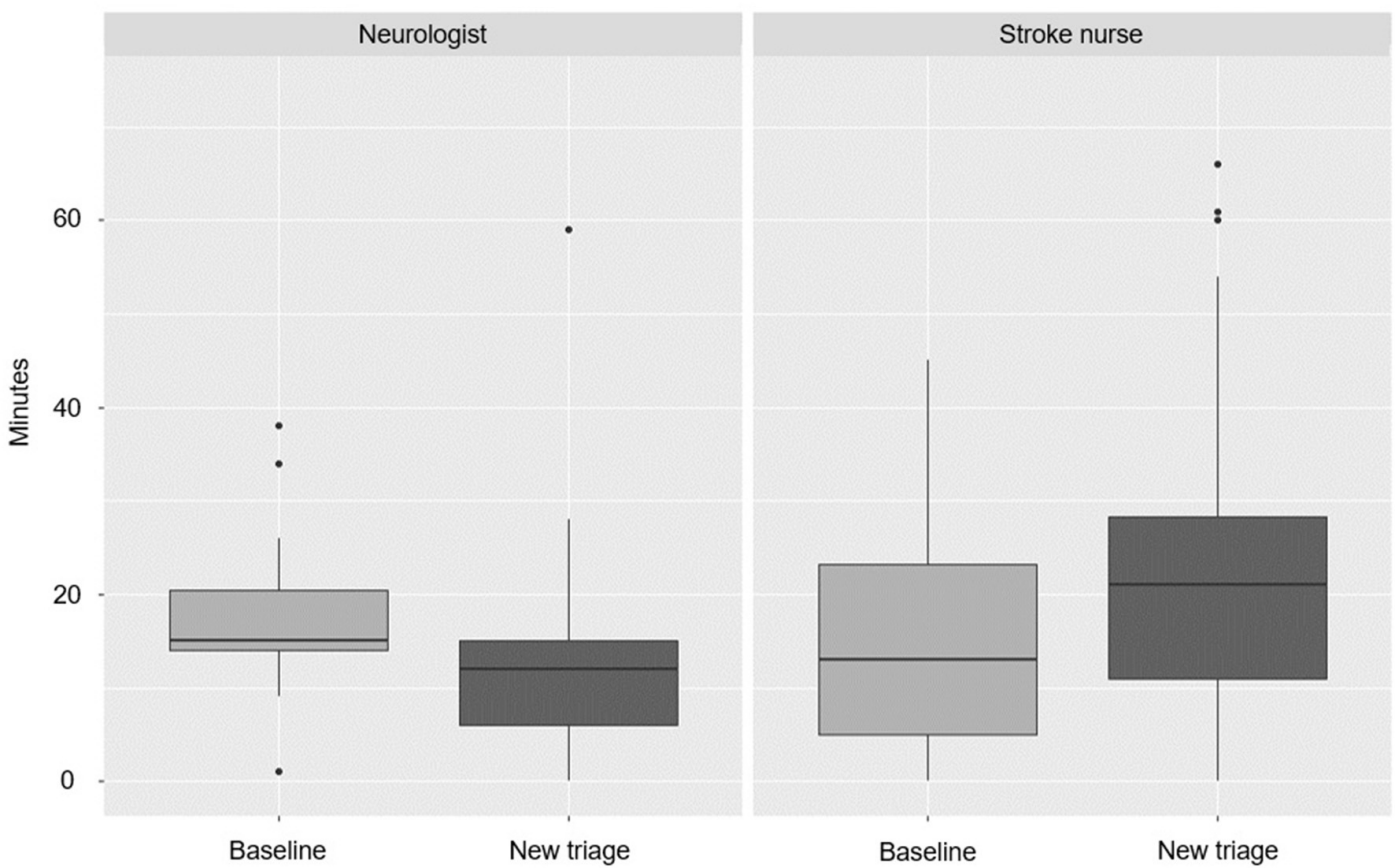
Time spent with patients per study phase and role (*p* = 0.036).

**Figure 5:**
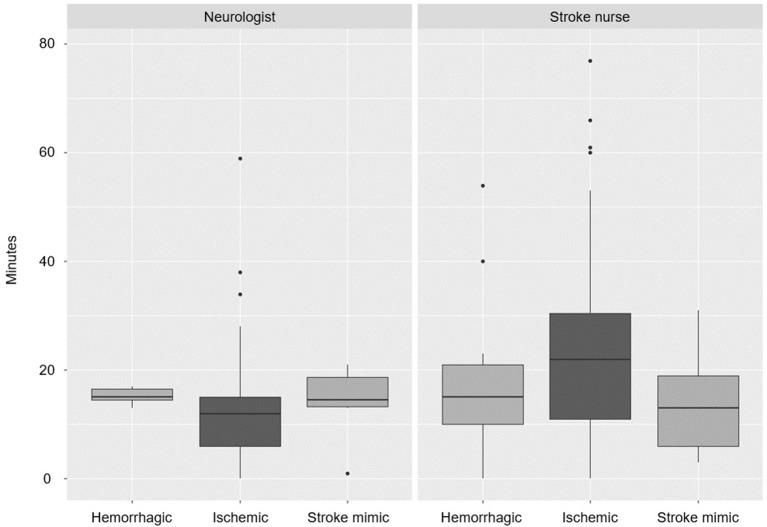
Time spent with patients per stroke type and role (*p* > 0.5).

After implementation of the new triage system, neurologists spent less time (from 15 ± 2.4 to 9 ± 1.3 min), whereas nurses spent significantly more time with patients (an increase from 13 ± 2.9 to 22 ± 5.1 min; *p* = 0.036, Figure 4). There was no influence of stroke type or of nonstroke diagnosis in the time staff spent with patients (Figure 5).

## Discussion

The RTLS was shown to measure the facility usage and patient–staff interaction times. The yield of automatic detection with complete accuracy was seen in 63% of cases over the course of 17 months.

### Facility Usage

Transport time is influenced by, among other factors, hospital layout. The significant pathway shift, including ER bypass, caused by the new triage system, had a visible impact on the time, while the patient is being moved through the hospital. Direct triage to neuroradiology resulted in a significant decrease in transport time by about 2 min (Figure 3). Time in holding waiting for a CT examination did not significantly vary over time and was in the order of 10–20 s in both the phases (Table 1). Usually, the CT rooms need to be prepared by the staff before usage (e.g., cleaning and setting up the machine). Therefore, we would expect that holding times would be shorter in phase I since transport time was longer and staff has more time to prepare the room. On the contrary, patients were almost never required to wait for a CT examination most likely because the staff at neuroradiology was well prepared and notified in advance in both the phases. Similarly, time in the CT room did not vary between the study phases (Table 1). The CT scan duration is fixed and the time corresponding to the actions performed in the CT room (e.g., administration of IVT or deciding where to transfer next) did not appear to be influenced by direct triage (*p* = 0.42). There was also a nonsignificant trend (*p* = 0.23) for longer times spent in neuroradiology for patients secondarily transferred from another hospital compared to directly triaged patients. The secondarily transferred patients were exclusively candidates for endovascular treatment after initial CT/CTA in the referring hospital. According to the previous local guidelines at our center (until 2019), they were re-evaluated with native CT, multiphase CTA, CTP, and discussed with the neurointerventionist before a decision was made for thrombectomy. This is to be expected since patients who are secondarily transferred usually have more severe strokes and large vessel occlusions (8, 9). This pathway was shown to be time-consuming in this study (107.4 min, neurointervention time included). On the other hand, some of these patients are re-evaluated with CT perfusion or multiphase CT and do not proceed to thrombectomy if the infarct size is too large, but this is also time-consuming (including time waiting for an available bed or transport back to the referring hospital) (10, 11). In our recently updated local guidelines, patients secondarily referred for thrombectomy with stroke onset times <6 h proceed directly to neurointervention in the absence of early neurological deterioration or complete recovery, thus decreasing the time spent in clinical and neuroradiological assessment. Median time spent in the CT room was slightly, but not significantly higher for hemorrhagic stroke patients. Most of these patients are evaluated with acute CTA, on-site consultation with the neurosurgeon, and eventual acute blood pressure management before transfer to another unit. Stroke mimics use slightly less CT room time in comparison, but still require clinical and imaging assessment before transfer, increasing the total time in neuroradiology to the same level of hemorrhagic stroke patients.

### Patient–Staff Interaction

Measuring how often staff and patients colocalize is potentially useful due to its ability to more realistically estimate resource allocation costs involved in certain workflows and procedures, a concept known as time-driven activity-based costing (12). In this study, the stroke nurse spent the most time with patients (Figure 5), which is expected since the stroke nurse has to attend to the patient through the care pathway. However, after changing triage of moderate-to-severe hemiparesis patients directly to neuroradiology, neurologists spent significantly less time with patients, while nurses spent more time as the study progressed. At baseline, neurologists spent more time with patients because the majority arrived at the ER, where they were examined by the neurologist, who also followed the patient during transport to the CT room. With direct triage, the preliminary diagnosis at the prehospital stage is more robust, since a dialog between the ambulance staff and receiving neurologist has already taken place, with the benefit of the region-wide EHR immediately available to the neurologist during the call. Upon arrival, the neurologist, thus, knows much more about the patient and the bedside history and examination take less time because of the time saved by previous teleconsultation and the EHR review. The need for keeping door-to-needle times short requires quick examination before decision on IVT or thrombectomy is made. In direct triage, stroke nurses also take over some of the tasks from ER nurses and need to stand by the patients longer also because they are more severely neurologically impaired. After implementing the new stroke pathway, stroke nurses spent a median time of 21 min in the first 5 months (compared to 13 min at baseline) and this still increased to 22 min in the last 7 months of the study, even if they had more experience and were trained in the new pathway. This likely reflects the increased number of tasks being performed and the increased stroke severity requiring more nursing. Interestingly, patient–staff interaction time was the same for stroke mimics and hemorrhagic stroke patients. Deciding on stroke mimic diagnosis and treatment for other disorders (e.g., epilepsy) is also time-consuming for the staff and the facility.

The RTLS offers an option for long-term assessments of bottlenecks in stroke care pathways instead of consuming staff and time resources for short-term audits, surveys, or shadowing exercises. Common bottlenecks in stroke pathways are ambulance dispatch and arrival to the correct hospital, distances from door-to-treatment (hospital layout, elevators, etc.), history taking, current medication check, team availability, patient history communication between teams, and resource management (imaging requests, CT room preparations, thrombolysis, anesthesia availability, and blood sampling). Typically, manually collected times during these exercises are not always registered immediately and often filled in later because the acute treatment has priority. This leads to a higher chance of misreporting times. The RTLS analytics, albeit only focusing on bottlenecks related to distance times, facility, and resource/staffing usage, can alleviate the burden of the staff to allow focus on treatment. The major advantage of this innovation is that it allows further description of time and staff logistics in more detail and at different areas where the stroke care is taking place, which cannot be analyzed from compact total door-to-needle or door-to-groin puncture times.

### Cost-Effectiveness

The installation costs of the RTLS are dependent on accuracy desired, number of rooms/zones to be covered, and use cases (for example, equipment, patients). The estimated infrastructure cost per room-level accuracy is about 500 Euro and the total cost for this study (equipment, personnel, installation, and training) was about 25,000 Euro. Data from the system can spare about 2–3 weeks of data collection, analysis, and reporting per year (conservative cost savings of about 3,000 Euro in salaries per year and releasing additional time for clinical work and production). With the current room installation, any clinical pathway from the ER to neuroradiology (e.g., neurotrauma, meningitis) can be analyzed by switching the tags to the different personnel and patient groups. Net turnover could be achieved after 3–5 years of usage. The results of this study also stressed the importance of having a backup nurse to the principal stroke nurse at neuroradiology, since the latter was busier with more tasks to perform, which has been implemented. Thus, reorganizing staff can lead to time savings in door-to-treatment times and to improve outcomes in the long term.

### Limitations

Several technical limitations resulted in missing data, which could be improved by changing the placement of patient tags and backup systems. Patient recruitment could not be made consecutively owing to refusal to consent and staffing rotation. Inferences based on characteristics such as age, sex, and stroke severity were not possible due to privacy restrictions and scope of study. The RTLS alone cannot make measurements of important metrics for stroke care pathways such as door-to-needle time. With only location data available, the closest metric that could be inferred would approximately be the time from patient arrival to leaving the CT room. We did not report the exact time when the neurologist and the stroke nurse arrived to the patient but, according to study design and usual clinical practice, the stroke nurse and the neurologist were deployed to the patient at the same time during the baseline and new pathway phases. Some exceptions may have occurred, but these would be expected in both the study phases and to be of short duration (for instance with busy, parallel stroke cases or telephone calls). Thus, the extra time spent by the nurse indicates that the nurse stayed longer with the patient, which is what we have observed.

## Conclusion

After changing the stroke triage system, median times of hospital arrival to leaving the CT room were cut by about 10 min. The RTLS was able to demonstrate that direct triage of moderate-to-severe hemiparesis patients increased stroke nurse–patient interaction times. The teleconsultation with the ambulance led to a decrease in the neurologist–patient interaction times. The RTLS can be useful for automatic identification of bottlenecks in stroke pathways and assist in staff management. Further technical improvements and interaction with the EHR can increase detection yield and workflow characterization in more detail.

## Data Availability Statement

The raw data supporting the conclusions of this article will be made available by the authors, without undue reservation.

## Ethics Statement

The studies involving human participants were reviewed and approved by Stockholm Regional Ethical Council (nr 2017/511-31/4). The patients/participants provided their written informed consent to participate in this study.

## Author Contributions

All authors listed have made a substantial, direct, and intellectual contribution to the work, and approved it for publication.

## Funding

This study received funding from Karolinska University Hospital and Philips Research.

## Conflict of Interest

AF and ED are employed by Philips Research. EL was previously employed by Philips Research and is now retired. Philips Research was involved in study design, equipment installation, data collection, and analysis, preparation of the manuscript and decision to submit for publication. The remaining authors declare that the research was conducted in the absence of any commercial or financial relationships that could be construed as a potential conflict of interest.

## Publisher's Note

All claims expressed in this article are solely those of the authors and do not necessarily represent those of their affiliated organizations, or those of the publisher, the editors and the reviewers. Any product that may be evaluated in this article, or claim that may be made by its manufacturer, is not guaranteed or endorsed by the publisher.
