# Supplementary figures and images for: Staff and Facility Utilization in Direct Patient Transfer to the Comprehensive Stroke Center: Testing a Real-Time Location System for Automatic Patient Pathway Characterization
Source: Front Neurol. 2021 Nov 24;12:741551. doi: 10.3389/fneur.2021.741551 (PMC8651566; doi:10.3389/fneur.2021.741551)

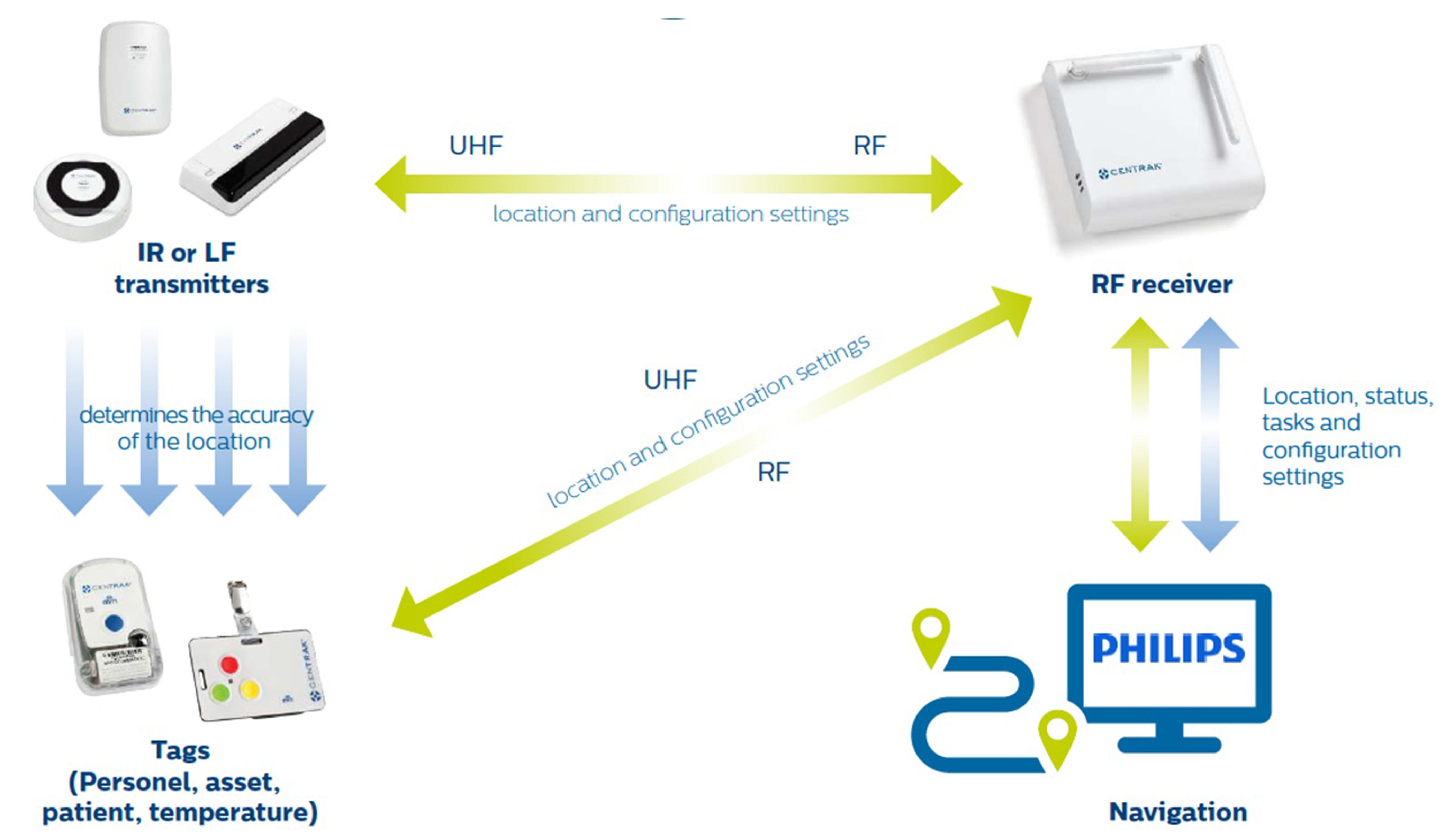

Supplement: Supplementary file 1 [file Image_1.JPEG]
